# Perceived social support among percutaneous coronary intervention patients over a long‐term follow‐up period

**DOI:** 10.1002/nop2.2087

**Published:** 2024-02-08

**Authors:** Outi Kähkönen, Leila Paukkonen, Hannu Vähänikkilä, Anne Oikarinen

**Affiliations:** ^1^ Department of Nursing Science, Faculty of Health Scicences University of Eastern Finland Kuopio Finland; ^2^ Research Unit of Health Sciences and Technology, Faculty of Medicine University of Oulu Oulu Finland; ^3^ Medical Research Center Oulu (MRC Oulu) Oulu Finland; ^4^ Northern Finland Birth Cohorts, Arctic Biobank, Infrastructure of Population Studies, Faculty of Medicine University of Oulu Oulu Finland

**Keywords:** percutaneous coronary intervention, social support

## Abstract

**Aim:**

To investigate perceived social support and the associated factors as well as the sources of social support among post‐percutaneous intervention patients over a long‐term follow‐up period.

**Design:**

An explanatory and descriptive survey with a six‐year follow‐up (STROBE Statement: Supplementary file 1).

**Methods:**

Baseline data (*n* = 416) were collected from Finnish patients in 2013, with follow‐up data collected from the same study group in 2019 (*n* = 154). The research employed the Social Support of Patients with Coronary Heart Disease self‐reported questionnaire. Data were analysed using descriptive statistics and multivariate methods.

**Results:**

In the acute phase, higher informational support was associated with lower LDL cholesterol and female gender and higher emotional support with working status. In long‐term follow‐up period, physical activity, younger age, normal cholesterol levels and previous percutaneous coronary intervention predicted higher informational support, regular participation in follow‐up sessions and relationship status predicted higher emotional support, and previous coronary artery bypass grafting, smoking, alcohol consumption, normal cholesterol and regular follow‐ups predicted higher functional support.

**Patient or Public Contribution:**

No Patient or Public Contribution.

## 1 INTRODUCTION

Atherosclerotic cardiovascular disease (ASCVD) incidence and mortality rates are declining in many countries in Europe, but they are still a major cause of morbidity and mortality causing about 1.9 million deaths yearly mainly corresponding with heart attacks and strokes (World Health Organization [WHO], 2021). These conditions were major causes of deaths in Europe being responsible 37% of all deaths, respectively (Eurostat, 2019).

Over the past few decades, major ASCVD risk factors have been identified. The most important way to prevent ASCVD is to promote a healthy lifestyle. Nevertheless, the prevalence of unhealthy lifestyles among the public is high (Visseren et al., 2021), and a large proportion of people at high ASCVD risk have poor control of risk factors: 18.1% of patients being smokers, 43.5% obese and 63.8% centrally obese, patient with hypertension 53% and dyslipidaemia 53.1% (Kotseva et al., 2020). Additionally, psychosocial factors, such as negative emotions, stress, depression and anxiety, low socio‐economic status and lack of social support, have been associated with morbidity and mortality of coronary heart disease (CHD; van Montfort et al., 2018). CHD is the most common manifestation of ASCVD, accounting for 49.2% of all incidents (Roth et al., 2020). The morbidity and mortality of CHD in various countries will continue to increase with the intensification of the aging process, which seriously threatens the health of the people and brings a heavy social and economic burden (Guo et al., 2020).

Regarding treatment options for CHD patients, percutaneous coronary intervention (PCI) is a common procedure for relieving obstruction in a stenotic coronary artery. The data demonstrate that PCI treatment has experienced exponential growth, with popularity still expanding worldwide (Knuuti et al., 2019). Thus, PCI is the most common invasive treatment option. For example, in Finland in 2020, slightly less than 16,000 PCIs and 1124 bypass operations were performed (Finnish Cardiac Society, 2020). However, the rapid growth of PCI treatment has not been sufficiently matched by the quality of care, counselling and social support provided to patients (Visseren et al., 2021). Following PCI, adherence to treatment requires both maintaining proper medication and lifestyle changes to prevent restenosis and other vascular events (Wang et al., 2020). Previous research has shown that approximately 15%–30% of post‐patients experienced restenosis less than 1 year after PCI (Alfonso et al., 2018). Therefore, the prevention and treatment of coronary heart disease are extremely important (Guo et al., 2020).

## 2 BACKGROUND

Although PCI effectively saves the lives of patients who experience CHD, the sudden occurrence of a cardiac event influences a patient's life in many ways (Pedersen et al., 2017). For example, patients may experience stress and uncertainty when their health status has suddenly changed (Doedee et al., 2021; van Montfort et al., 2018).

The concept of social support is multidimensional and can be incorporated into a larger context termed social capital, where social support and social networks are parts (Drageset, 2021). Social support described in different ways. One commonly used definition is based on the Theory of Social Support presented by Cohen and Wills (1985). According to the theory, social support can be defined as a dynamic interpersonal process that includes the interactive exchange of information that changes across different contexts (Cohen & Wills, 1985). Social support consists of emotional, informational and functional support. The theory defines the three different aspects of social support as follows: emotional support includes listening, encouragement, the provision of care and appreciating trust; informational support comprises receiving information, recommendations and feedback; and functional support consists of healthcare professionals offering time, counselling and assistance to people who are having trouble with coping with their illness and/or current life situation (Cohen & Wills, 1985). Thus, social support refers to social network characteristics that help individuals to cope with everyday life, especially in the face of critical situations. These social network characteristics primarily included a variety of assistance from family, neighbours, friends and other community members (Drageset, 2021).

The importance of social support is emphasized during stressful life events, with a social network offering a sense of security, love and community (Clayton et al., 2019; Hu et al., 2022; Li et al., 2019). Earlier research has shown that stressful life events and perceived social support have independent significant effects on health‐related quality of life (HRQoL) in CHD patients (Djekic et al., 2020; Hu et al., 2022; Li et al., 2019). Also, Pushkarev et al. (2019) reported that the level of social support was associated with age and gender and significantly and independently affected CHD patients' risk of death after PCI.

Support from nurses during the recovery after PCI is a key predictive factor of higher perceived health (Kähkönen et al., 2021; Shi et al., 2022). The amount of hospitalization required after PCI has significantly decreased in recent years, and this naturally affects the amount of social support that patients receive in the hospital (Pushkarev et al., 2019; Tuomisto et al., 2018). A low level of social support is an independent risk factor for CHD in healthy individuals and negatively affects the CHD prognosis (Mondesir et al., 2018; Pushkarev et al., 2019; Zhang, 2021). As such, individuals who lack social support have a higher prevalence of traditional cardiovascular risk factors (Pedersen et al., 2017). Furthermore, there is evidence that low levels of social support are strongly connected to higher mortality in CHD patients (Pushkarev et al., 2019; Zhang, 2021). It has been suggested that social support benefits adherence to a healthier lifestyle (Hu et al., 2022; Shi et al., 2022); for example, people who experience higher levels of social support are more physically active (Resurrección et al., 2019; Shi et al., 2022), and they demonstrate better adherence to the self‐care recommendations (Shi et al., 2022). Social support is connected to both mental health (Sun et al., 2022; Thagizadeh et al., 2022; Zhang, 2021) and an individual's perceived physical health (Kim, Kim, et al., 2019; Kim, Lim, et al., 2019), as well as related to certain physiological factors such as cardiac responses to acute stress and blood pressure control (Thagizadeh et al., 2022), stimulation of the autonomic nervous system and plaque‐destabilizing factors (Huang et al., 2022). A lack of social support has related to anxiety and depression (Hu et al., 2022; Thagizadeh et al., 2022; Zhang, 2021), which may affect a person's health indirectly through unhealthy behaviours or directly via physiological mechanisms (Freak‐Poli et al., 2022; Hu et al., 2022; Zhang, 2021). For this reason, versatile support is needed during post‐PCI recovery. (Hu et al., 2022). Perceived social support has been the subject of interest in post‐PCI patients (e.g Clayton et al., 2019). However, research‐based knowledge about perceived social support and its sources is scarce over a long‐term follow‐up. Therefore, this study aimed to investigate perceived social support and the associated factors, as well as the sources of social support over a long‐term follow‐up among post‐PCI patients.

## 3 THE STUDY

### 3.1 Aim

#### 3.1.1 Study design

The study utilized an explanatory and descriptive survey with six‐year follow‐up. The study was reported according to the STrengthening the Reporting of OBservational Studies in Epidemiology [STROBE] Statement: guidelines for reporting observational studies (File S1).

### 3.2 Settings and participants

The CHD patients, who were between 18 and 75 years of age and had no diagnosed memory disorders, were recruited for the study from January to December 2013 from medical wards at two university hospitals and three central hospitals 4 months after an elective or acute PCI procedure (Nikolopoulou, 2022). The participants were recruited using convenience sampling, meaning that every patient who was treated by PCI and met the inclusion criteria was equally entitled to participate. Thus, all patients meeting the inclusion criteria were invited to participate in the study.

A total of 572 patients met the inclusion criteria at the baseline measurement point. Participants were informed about the background and objectives of the study, after which 520 of the patients gave informed consent to participate in the study. This sample size was large enough to detect statistical significance with a power of 80% and a significance level of 0.05 given relatively small correlations (0.14). This sample size could be expected to detect between 7% and 13% of the differences between groups. A total of 416 patients, representing a response rate of 81%, completed the postal questionnaire 4 months after PCI. Of these patients, 352 (84.6%) gave their permission to participate in the follow‐up study. The follow‐up study was conducted 6 years after PCI in June 2019, when 154 (43.8%) of the patients who had completed the initial survey responded to the follow‐up questionnaire. Participants received a questionnaire with a stamped, addressed return envelope, as well as detailed written information about the study purpose and objectives and contact information for the researcher.

### 3.3 Measurements

The Social Support of People with Coronary Heart Disease (SSCHD) instrument was used to measure perceptions of social support among CHD patients at the baseline in 2013, as well as in the follow‐up study in 2019. The instrument was self‐developed and used in Finnish language for the baseline study. It is based on Cohen and Wills' (1985) theory of social support, which comprises three dimensions of social support: emotional support; informational support; and functional support (Cohen & Wills, 1985).

SSCHD instrument includes seven sum variables concerning informational support, four sum variables concerning emotional support and three sum variables concerning functional support (Table 1). The dimension related to informational support contained the following items: advice on physical exercise after PCI; advice on risk factors; knowledge of their own risk factors; advice on how to behave when experiencing chest pain; information on medication; and information on the continuum of care and rehabilitation. The dimension measuring emotional support included perceived support from other cardiac patients, family and friends, along with the patients' perceived importance to their next of kin. The dimension measuring functional support included the opportunity to ask questions about issues of concern, feeling support and care, and cooperation with healthcare professionals.

**TABLE 1:** Factor loadings, eigenvalues of the factors and Cronbach's alpha values of the sum variables of perceived social support.

SSCHD instrument	Factor loading	Eigenvalue of the factors	Cronbach's alpha
			Total 0.78
Factor 1: Informational support	0.50–0.72	5.6	0.84
Information on the continuum of care and rehabilitation			
Information on medication			
Information on physical exercise after PCI			
Advice on own risk factors			
Information on how to act in the case of chest pain			
Information on CHD			
Knowledge of own risk factors			
Factor 2: Emotional support	0.34–0.75	1.5	0.60
Support from friends			
Support from family			
Importance to next of kin			
Support from other patients			
Factor 3: Functional support	0.81–0.86	1.2	0.90
Healthcare professionals care about patient coping with CHD			
Opportunity to ask healthcare professionals about issues of concern			
Support from healthcare professionals			

Abbreviations: CHD, coronary heart disease; PCI, percutaneous coronary intervention.

The participants answered each item using a five‐point Likert‐type scale ranging from definitely disagree (1) to definitely agree (5). The formatted sum variables were coded into two categories as follows: values 3.9 and below were combined and assigned a value of 1, which represents a low level of social support; values ranging from 4.0 to 5.0 were combined and assigned a value of 2, which represents a high level of social support.

Additionally, information about patients' sociodemographic (gender, age, marital status, working status, profession, length of education), health behavioural (physical activity, vegetable and alcohol consumption, smoking habits) and disease‐specific (systolic blood pressure, total cholesterol, LDL cholesterol, prior PCI or GABG, duration of CHD, blood pressure medication, cholesterol medication) factors was collected. Furthermore, the patients were asked to complete questions related to secondary prevention (e.g., regular follow‐up appointments and participation in cardiac rehabilitation).

Regarding the SSCHD instrument validity, Cronbach's alpha values were calculated to evaluate the internal consistency of sum variables; these values indicated week internal consistency for the dimension of emotional support (α 0.60) and satisfactory internal consistency for functional support (α 0.90) and informational support (α 0.84). The Cronbach's alpha value for the entire instrument was 0.78, which represents acceptable internal consistency (Polit & Beck 2017). In addition, three experienced nurses of cardiac patients and 15 patients with CHD and prior PCI evaluated the face validity of the questionnaire. Changes were made based on the nurses' and patients' comments to ensure intelligibility and usability of the instrument.

### 3.4 Statistical analyses

Frequencies and descriptive statistics (amounts and percentages) were computed for the variables to describe the respondents' sociodemographic, health behavioural and disease characteristics. The associations between perceived social support and the background variables were analysed by cross‐tabulations, and the statistical significance of these associations was tested using chi‐squared tests. In the case that the chi‐squared test was not appropriate (no more than 20% of cells should have a value <5), Fisher's exact test was used (Burns & Grove 2009; Polit & Beck, 2017). Multivariate logistic regression was used to confirm the standardized predictors of social support. The variables were entered into the model using forward stepwise selection. The threshold for statistical significance was set as *p* < 0.05 (Burns & Grove 2009; Polit & Beck, 2017). Respondents were also asked to identify every source of informational support in the open‐ended questions, after which the reported sources of support were quantified.

### 3.5 Ethical considerations

Approval for the study was obtained from each research centre and the Ethical Review Board of the University Hospital of XX (Ref. XX/XX). In accordance with the Declaration of Helsinki, participants received verbal and written information about the study, which was provided by a Registered Nurse, before signing the consent forms and being discharged. This information included the purpose and procedures of the study and stated the voluntary nature of participation, including the option to withdraw at any point. Research ethics approval was obtained from the ethical committees of each participating research centre. Informed consent was obtained from all of the participants included in the study.

## 4 RESULTS

### 4.1 Sample characteristics

The 154 respondents, of which 118 (86.6%) were male, had an average age of 68.5 + 7.0 years. At the baseline time point, 46 (29.8%) of the respondents had already undergone a previous PCI, while 21 (13.6%) had undergone a CABG. Additionally, 48 (31.2%) of the respondents had suffered an AMI. Between the current PCI and the six‐year follow‐up time point, three patients (1.9%) had undergone another PCI, one (0.6%) had undergone an CABG, and two patients (1.3%) had suffered an AMI. Details of the respondents' clinical characteristics are presented in Table 2.

**TABLE 2:** Disease‐specific, sociodemographic and health behavioural characteristics of patients 4 months and 6 years after percutaneous coronary intervention.

Factor	Baseline 2013 (*n* = 154)	Follow‐up (*n* = 154)
	*n* (%), mean + SD	*n* (%), mean + SD
SOCIODEMOGRAPHIC FACTORS		
Gender, male	118 (76.6)	118 (86.6)
Age (years)	63.3 ± 7.5	68.5 ± 7.01
Marital status		
Married/close relationship	128 (83.1)	117 (76.0)
Working status		
Employed	35 (25.4)	23 (14.9)
Unemployed	9 (6.5)	5 (3.6)
Retired	92 (66.7)	120 (78)
Part time retired/at long sick leave	2 (1.4)	6 (3.65)
HEALTH BEHAVIOURAL FACTORS		
Vegetable consumption dl/day	2.3 + 1.3	2.5 + 1.1
Smoking	30 (19.6)	14 (8.1)
Physical activity > 120 min per week	69 (45.4)	63 (40.9)
Alcohol consumption, <2 portions	67 (49.3)	94 (63.9)
DISEASE‐SPECIFIC FACTORS		
Systolic blood pressure		
≤ 139 mmHg	100 (64.9)	107 (69.5)
Hypertension	42 (27.3)	41 (26.6)
Did not know blood pressure	12 (7.8)	6 (3.9)
Total cholesterol		
≤ 4.5 mmo1/L	68 (44.2)	74 (48.1)
Hypercholesterolaemia	21 (13.6)	15 (9.7)
Did not know total cholesterol	65 (42.2)	65 (42.2)
LDL cholesterol		
≤ 1.8 mmol/L	46 (29.9)	34 (22.1)
Hypercholesterolaemia	52 (33.8)	48 (31.2)
Did not know LDL cholesterol	56 (36.4)	72 (46.8)
Prior PCI	46 (29.8)^a^	3 (1.9)^b^
Prior CABG	21 (13.6)^a^	1 (0.6)^b^
AMI	48 (31.2)^a^	2 (1.3)^b^
Duration of CHD	5.0 ± 8.1	9.7 ± 7.6
Blood pressure medication	113 (78.5)	123 (80.4)
Cholesterol medication	NA	136 (90.1)
SECONDARY PREVENTION		
Regular follow‐up	NA	113 (73.9)
Participation in cardiac rehabilitation	NA	47 (30.9)

*Note*: The values denote mean ± standard deviation or *n* (%).

Abbreviations: AMI, acute myocardial infarction; CABG, coronary artery bypass grafting; CHD, coronary heart disease; PCI, percutaneous coronary intervention.

^a^ Prior to index PCI.

^b^ Between 2013 and 2019.

### 4.2 Perceived social support 4 months and 6 years after PCI

The majority of the respondents reported high levels of overall social support (92.7%), as well as informational (96.1%), emotional (97.6%) and functional support (86.1%), during the acute phase of care, that is, 4 months after PCI. Moreover, perceived social support remained high in the long‐term follow‐up period (6 years after PCI), with 85.2% of the respondents reporting high levels of social support. Furthermore, 75.1%, 73.4% and 62.1% of the respondents reported high levels of informational, emotional and functional support, respectively, at the long‐term follow‐up time point. The mean scores of all dimensions of social support showed statistically significant changes across the four‐month and six‐year post‐PCI measurement points (Table 3): informational support 4.56 vs. 4.22, *p* = < 0.001; emotional support 4.44 vs. 4.20, *p* = 0.002; and functional support 4.25 vs. 3.75, *p* = <0.001.

**TABLE 3:** Differences between the groups regarding perceived social support (informational, emotional and functional) 4 months and 6 years after PCI, as calculated by Wilcoxon test.

Perceived social support	Mean	25th–75th percentile	*p*
Informational support			
Baseline 2013	4.56	4.43–5.00	<0.001
Follow‐up 2019	4.22	3.98–4.86	
Emotional support			0.002
Baseline 2013	4.44	4.00–5.00	
Follow‐up 2019	4.20	3.75–4.75	
Functional support			<0.001
Baseline 2013	4.25	4.00–5.00	
Follow‐up 2019	3.75	3.00–4.67	

At the four‐month measurement point, higher informational support was associated with lower LDL cholesterol levels and female gender (Table 4). Participation in regular follow‐ups, physical activity, younger age, normal total cholesterol levels and having only one PCI were predictors of higher perceived informational support at the six‐year follow‐up measurement point (Table 5). Higher emotional support was associated with working status at the four‐month measurement point, while participation in regular follow‐ups and close relationships were significantly associated with higher emotional support 6 years after PCI. An analysis of the four‐month time point data did not reveal any association between functional support and the measured background factors. On the other hand, previous CABG, smoking status and participation in regular follow‐ups during secondary prevention were associated with higher functional support during the long‐term follow‐up period.

**TABLE 4:** Associations between perceived social support and sociodemographic, disease‐specific and health behavioural background factor instrument 4 months after percutaneous coronary intervention. SSCHD mean value and mean difference with 95% confidence interval to the reference group from multivariate analysis of variance (*n* = 154).

Variable	B (95% CI)	*p*
Informational support		
LDL cholesterol	−0.54 (−0.86/−0.22)	0.001
Gender	0.35 (−0.86/−0.22)	0.001
Emotional support		
Working status	−0.11 (−0.22/−0.01)	0.04
Functional support		
None		

*Note*: Confidence intervals to the reference group from multivariate analysis of variance (*n* = 154).

**TABLE 5:** Associations between perceived social support and sociodemographic, disease‐specific and health behavioural background factors 6 years after percutaneous coronary intervention. SSCHD mean value and mean difference with 95% confidence interval to the reference group from multivariate analysis of variance (*n* = 154).

Variable	B (95% CI)	*p*
Informational support		
Regular follow‐ups in secondary prevention	0.78 (0.42/1.15)	<0.001
Physical exercise	0.49 (0.20/0.77)	0.001
Previous PCI	0.42 (0.10/0.73)	0.01
Age	−0.02 (−0.04/−0.02)	0.03
Total cholesterol	−0.64 (−1.02/−0.26)	0.001
Emotional support		
Regular follow‐ups in secondary prevention	0.50 (0.27/0.73)	<0.001
Relationship	−0.53 (−0.77/−0.30)	<0.001
Functional support		
Previous CABG	0.92 (0.36/1.47)	0.002
Smoking	0.93 (0.09/1.18)	0.03
Regular follow‐ups in secondary prevention	0.74 (0.15/1.33)	0.02
Age	−0.05 (−0.08/−0.02)	0.002
Alcohol consumption	−0.54 (−1.02/−0.06)	0.03
Total cholesterol	−0.08 (−1.38/−0.18)	0.01

Abbreviations: 95% CI, 95% confidence interval; B, mean difference; SSCHD, Social Support of People with Coronary Heart Disease.

### 4.3 Sources of perceived social support

Although patients reported high levels of informational support during care, there were differences in perceived informational support between the four‐month and six‐year post‐PCI measurement points. An inspection of the dimensions of informational support (Table 6) identified knowledge of personal risk factors (98.2% vs. 95.1%) and advice on own risk factors (98.2% vs. 95.1%) as indicated as the areas with the highest level of perceived support; this was true for both the four‐month and six‐year follow‐up time points. The results for various dimensions of informational support were information on CHD as a disease (96.4% vs. 92.7%), advice on physical exercise (95.2% vs. 88.9%), advice on what to do in the event of chest pain (93.5% vs. 90.1%) and advice on medication (90.9% vs. 88.2%), with the percentages indicating the shares of patients who reported high levels of support. The weakest area of informational support was the continuum of care and rehabilitation, with 86.7% and 64.4% of the patients reporting high levels of support in the four‐month and six‐year measurement points, respectively. Concerning emotional support, respondents assessed support from family (97.6% vs. 97.0% for the four‐month and six‐year measurement points, respectively) the highest and support from other patients (72.3% vs. 63.9% for the four‐month and six‐year measurement points, respectively) the weakest. Concerning functional support, the respondents felt that they had the most support from healthcare professionals (93.2% vs. 99.2% for the four‐month and six‐year measurement points, respectively) and the least support to ask about issues of concern (84.8% vs. 78.5% for the four‐month and six‐year measurement points, respectively).

**TABLE 6:** Sources of informational support among post‐percutaneous coronary intervention patients 4 months after PCI (*n* = 154).

Source of informational support	Information on CHD	Advice on own risk factors	Advice on conduct in situations of chest pain	Advice on medication	Advice on physical exercise	Information on continuum of care and rehabilitation	Total
	*n* (%)^a^	*n* (%)^a^	*n* (%)^a^	*n* (%)^a^	*n* (%)^a^	*n* (%)^a^	*n* (%)^a^
Physician	108 (48.2)	111 (50.7)	97 (51.1)	106 (56.4)	89 (51.4)	63 (44.7)	574 (50.3)
Nurse	79 (35.3)	89 (40.6)	82 (43.1)	58 (30.8)	59 (34.2)	63 (44.7)	430 (37.7)
Internet	16 (7.1)	7 (3.2)	4 (2.1)	3 (1.6)	3 (1.7)	5 (3.5)	38 (3.3)
Booklet and media	6 (2.7)	3 (1.4)	2 (1.1)	5 (2.7)	4 (2.3)	7 (4.9)	27 (2.4)
Rehabilitation	6 (2.7)	4 (1.8)		1 (0.5)			11 (1.0)
Physiotherapist	2 (0.9)			11 (5.8)	16 (9.3)	3 (2.1)	38 (3.3)
Pharmacy	1 (0.5)	1 (0.5)	1 (0.5)	4 (2.2)	2 (1.1)		9 (0.8)
Family/friends	6 (2.6)	4 (1.8)	4 (2.1)				14 (1.2)
Total	224 (100)	219 (100)	190 (100)	188 (100)	173 (100)	141 (100)	1141 (100)

^a^ Numbers and percentages of all mentioned sources of informational support.

The most commonly mentioned sources of informational support (Table 6 baseline vs. Table 7 follow‐up) were support from physicians (50.3% vs. 53.8% for the four‐month and six‐year measurement points, respectively) and support from nurses (37.7% vs. 32.1% for the four‐month and six‐year measurement points, respectively). Additionally, respondents reported receiving informational support from the Internet (3.3% vs. 3.1% for the four‐month and six‐year measurement points, respectively), physiotherapists (3.3% vs. 0.2% for the four‐month and six‐year measurement points, respectively), booklet and media (2.4% vs. 1.5% for the four‐month and six‐year measurement points, respectively), during rehabilitation (1.0% vs. 3.6% for the four‐month and six‐year measurement points, respectively) and at the pharmacy (0.8% vs. 11.5% for the four‐month and six‐year measurement points, respectively). Regarding informational support from physicians, advice on medication (56.4% vs. 70.9% for the four‐month and six‐year measurement points, respectively) received the highest share of positive responses from the patients, while advice on the continuum of care and rehabilitation was most the common form of informational support received from nurses (44.7% vs. 45.3% for the four‐month and six‐year measurement points, respectively).

**TABLE 7:** Sources of informational support among post‐percutaneous coronary intervention patients 6 years after PCI (*n* = 154).

Source of informational support	Information on CHD	Advice on own risk factors	Advice on how to act in the case of chest pain	Advice on medication	Advice on physical exercise	Information on the continuum of care and rehabilitation	Total
	% (*n*)^a^	% (*n*)^a^	% (*n*)^a^	% (*n*)^a^	% (*n*)^a^	% (*n*)^a^	% (*n*)^a^
Physician	100 (48.7)	100 (55.6)	85 (55.6)	105 (70.9)	86 (51.8)	52 (49.1)	528 (53.8)
Nurse	58 (28.2)	57 (31.8)	53 (34.4)	28 (18.8)	71 (42.8)	48 (45.3)	315 (32.1)
Internet	17 (8.3)	6 (3.3)	4 (2.6)	2 (1.4)	1 (0.6)		30 (3.1)
Booklet and media	9 (4.4)	3 (1.7)	1 (0.7)		1 (0.6)	1 (0.9)	15 (1.5)
Rehabilitation	11 (5.4)	8 (4.4)	5 (3.3)	2 (1.4)	4 (2.4)	5 (4.7)	35 (3.6)
Physiotherapist	2 (1.0)	1 (0.5)					2 (0.2)
Pharmacy	3 (1.5)	1 (0.5)	1 (0.7)	9 (6.1)	1 (0.6)		15 (1.5)
Family/friends	5 (2.5)	4 (2.2)	4 (2.76)	2 (1.4)	2 (1.2)		41 (4.2)
Total	205 (100)	180 (100)	154 (100)	148 (100)	166	106 (100)	981 (100)

^a^ Numbers and percentages of all mentioned sources of informational support.

## 5 DISCUSSION

To the best of our knowledge, only a few previous studies have investigated perceived social support among post‐PCI patients over the long term. Thus, the six‐year follow‐up period described in this study provides valuable information for developing patient‐centred care and counselling.

The patients who participated in this study reported high levels of social support after PCI, which agrees with the findings of Li et al. (2019) and Pushkarev et al. (2019). However, the patients experienced significantly lower levels of social support during the long‐term follow‐up period than in the acute phase of care. This result is clinically important because there is strong evidence that low social support is an independent risk factor for CHD (Mondesir et al., 2018; Zhang, 2021) and seems to negatively influence CHD prognosis (Pushkarev et al., 2019).

Post‐PCI patients might experience a mismatch between their expectations and reality because of the effectiveness of treatment, which leads to rapid discharge (Valaker et al., 2017). Previous evidence shows that although CHD patients receive information about risk factors, support is critical for adherence to treatment (Kähkönen et al., 2020; Nicolai et al., 2018; Shi et al., 2022). Patients often perceive the PCI procedure as a curative treatment and that their illness is acute rather than chronic (Ashour et al., 2020); these perceptions have been shown to be associated with adverse outcomes (Thagizadeh et al., 2022) and poor adherence to treatment (Kähkönen et al., 2017).

Our results showed that perceived social support among CHD patients changes over time. Regarding the dimensions of social support, higher informational support in the acute phase of care was associated with lower LDL cholesterol levels. This is noteworthy because high LDL cholesterol levels are a major risk factor for the progression of CHD after PCI (Visseren et al., 2021). Thus, the provision of informational support in the acute phase after PCI improves the possibility of achieving treatment goals (Hu et al., 2022; Nicolai et al., 2018; Sun et al., 2022; Valaker et al., 2017) and may help patients accurately understand their illness and adapt to a healthier lifestyle; this was observed, for example, in the study by Pushkarev et al. (2019) and Hu et al. (2022). There was a higher share of female patients in the group that reported poor perceptions of social support than in the group with better perceptions of social support; this agreed with what was reported by Djekic et al. (2020).

Over the long‐term follow‐up period, higher perceived informational support was associated with key risk factors (physical activity and cholesterol levels, among others) that are linked to the prevention of new cardiac events (Shi et al., 2022; Zhang, 2021). Our results showed that older people experience lower levels of informational support in the long‐term follow‐up period relative to younger patients. As previously stated by Pushkarev et al. (2019), adequate informational support is particularly important for older post‐PCI patients, as age has been established as an independent risk factor for CHD (Sun et al., 2022; Visseren et al., 2021). The proper treatment of risk factors and understanding CHD as a life‐long chronic disease prevents the progression of CHD (Ashour et al., 2020; Knuuti et al., 2019; Visseren et al., 2021) and could be expected to improve patients' health‐related quality of life (Hu et al., 2022; Li et al., 2019).

An interesting result linked to informational sources was that the respondents felt as though they received more informational support from doctors than nurses across every dimension of informational support. Informational support is a key feature of counselling, which is a core responsibility of nurses, so the result does not reflect the goals of nursing and partially contradicts earlier research data; for example, it has been found that nursing‐led interventions seem to improve various aspects of post‐PCI problems, for example, self‐management, lifestyle modifications, psychological well‐being and quality of life (Shi et al., 2022; Zhang & Qi, 2021).

The finding that patients received more informational support from doctors than from nurses could have diverse explanations. According to Lie et al. (2022), patients commonly forget or misunderstand 40%–80% of the information that they receive. Moreover, they may also prioritize key items of information, that is, those that enable and motivate certain behaviours (Lie et al., 2022). As such, it may be that the nurses provided counselling in the context of treatment interventions, and the patient did not consider this to be counselling, but rather conversation on a general level. Instead, physicians often meet patients face‐to‐face and focus on topics that are clearly related to the patient's medical situation. Thus, it is important to pay attention to the effectiveness of various forms of information provision (Kim, Kim, et al., 2019; Kim, Lim, et al., 2019; Lie et al., 2022; Nicolai et al., 2018).

In this study, a clinically important result was that patients who had regular primary care follow‐ups reported higher informational support than patients who rarely attended follow‐ups. The patient's increased knowledge is the central goal in nursing interventions after PCI (Hu et al., 2022). Long‐term nursing intervention can effectively improve the self‐management ability of patients after PCI, as seen in the result by Hu et al. (2022). The reason may be that long‐term nursing intervention can attend to all aspects of the patient's cognition, behaviour, humanities and social support in order to optimize the patient's physical, psychological, social and other functions and to effectively improve the patient's levels of health knowledge and self‐management. However, a concerning result is that information on the continuum of care and rehabilitation was the weakest part of informational support. The problem may lie in the continuity of care. There is a lack of a systematic treatment pathway, while treatment monitoring, participation in cardiac rehabilitation and secondary prevention are mainly considered the patient's own responsibility. As such, patients have to coordinate their own care, as was also seen in this study – only about three‐quarters of the respondents attended follow‐up visits and only a third participated in cardiac rehabilitation. In line with our results, Valaker et al. (2017) reported that post‐PCI patients experience discontinuity of care regarding discharge planning, follow‐up appointments with a general practitioner and access to cardiac rehabilitation. This means that patients visit various healthcare professionals at different organizations, which can lead to the fragmentation of care. The importance of regular follow‐up visits and adequate informational support is undeniable (Hu et al., 2022); for example, Ashour et al. (2020) provided evidence that patients experience less symptoms, have better perceptions of how the disease influences their life, and a more positive emotional response following PCI. Thus, healthcare professionals should encourage post‐PCI patients to get involved in their counselling, that is, enrol in periodic follow‐up meetings to promote positive changes and maintain their health and well‐being (Ashour et al., 2020; Hu et al., 2022; Zhuo et al., 2021).

The results of this study indicated that, during the acute phase of care, patients who were actively in the workforce experienced higher perceived emotional support than patients who were unemployed or retired. Over the long‐term follow‐up period, patients who reported close personal relationships or participated in regular follow‐ups experienced higher emotional support than other patients. These results reinforce the perspective that the counselling process should be more patient‐centred (Langberg et al., 2019; Sun et al., 2022; Valaker et al., 2017; van Montfort et al., 2018) and that family members of patients undergoing PCI should be involved in the care process (Köhler et al., 2017; Tuomisto et al., 2018; Zhuo et al., 2021). This means that healthcare professionals should ensure that post‐PCI patients who do not have close relationships receive adequate support, for example, peer support. Patients without a partner are at an increased risk of having an acute cardiac event regardless of gender and age, and their prognosis is worsened in the case of an acute cardiac event (Blakoe et al., 2022). Previous research has also shown that being in a close relationship may encourage patients to adhere a healthy lifestyle; thus, close relationships may have a protective effect on CHD prognosis, as mentioned by Köhler et al. (2017), Blakoe et al. (2022) and Freak‐Poli et al. (2022).

An interesting finding of the present study was the significant association between higher functional support and regular secondary prevention follow‐ups over the long term (i.e., 6 years). A cause for concern is that the weakest part of functional support – as well as all dimensions of social support – was patients’ opportunity to ask about issues of concern. This contradicts the principles of patient‐centred care and the results from Ayton et al. (2018), who indicated that an opportunity to voice concerns and ask questions are important parts of a patient's participation in care. In other words, this approach would ensure that the patient's needs are met because individualized care promotes patient participation, while active communication may further improve adherence to care (Paukkonen et al., 2021). These results are in line with the Current Care Guidelines, which recommend shared decision‐making between the patient and healthcare professional based on individual patient characteristics. Secondary prevention requires an integrated, interdisciplinary approach that includes inputs from several disciplines and areas of expertise. Moreover, care should be patient‐ and family‐centred, and each of the core components of prevention and rehabilitation, including lifestyle modification, psychosocial factors, risk factor treatment and social determinants, should be addressed (Knuuti et al., 2019; Visseren et al., 2021).

### 5.1 Limitations of the study

We acknowledge that the present study included some limitations. First, self‐reported data collection methods always include a risk of the social desirability effect in that patients provide answers that they think are favourable instead of truthful. In addition, the use of a disease‐specific instrument would have added value to the results of the study. The second limitation is related to bias associated with the recruitment process in 2013. Patients are generally discharged 24 h after PCI. Due to this rapid turnover, there is a risk that some patients who met the inclusion criteria for the study may have been overlooked. At the baseline (four‐month) measurement point, participants were asked permission to contact them regarding the follow‐up study, with 352 of the 416 patients (84.6%) giving informed consent. However, the final response rate after 6 years was 43.8% (*n* = 154). This is worrying, because it has been shown that patients who demonstrate good adherence to treatment are more likely to respond to questionnaires than patients who are less successful at adhering to treatment; this could have biased the presented results. Another limitation is that the results were analysed at the group level according to the research plan. Thus, the presented results should be generalized with caution.

## 6 CONCLUSIONS

Both measurement periods (4 months and 6 years after PCI) revealed relatively high levels of perceived social support among patients. The results suggest that perceived social support changes over time, that is, social support was significantly lower 6 years after PCI than 4 months after PCI. Thus, access to social support should be ensured in the long‐term follow‐up period of secondary prevention.

Regarding the different dimensions of social support, higher informational support was associated with lower LDL cholesterol levels and female gender during the acute care period and with regular follow‐ups, physical activity, young age, normal cholesterol levels and previous PCI over the long‐term follow‐up period. Furthermore, higher emotional support was predicted by working status during acute care and by regular participation in follow‐up sessions and relationship status over the long‐term follow‐up period. Higher functional support was predicted by previous GABG, smoking, regular participation in follow‐up sessions, alcohol consumption and cholesterol levels 6 years after PCI.

## 7 RELEVANCE TO CLINICAL PRACTICE

Social support is essential for patients who have undergone PCI and should be actively offered during different phases of the recovery process. The need for social support does not diminish following percutaneous coronary intervention, but it does change over time. The importance of social support following percutaneous coronary intervention is undeniable; this means that patients should be afforded access to support through regular follow‐ups and continuous care. Patient‐centred, individualized care, which includes informational, emotional and functional support, is important to ensuring good long‐term clinical outcomes.

## AUTHOR CONTRIBUTIONS

OK completed the data collection and analysis. OK and AO drafted the first version of the manuscript with feedback from all of the authors. The manuscript was then revised by all of the authors (HV, LP, AO), with OK taking the main responsibility for writing the manuscript. All of the authors approved the final version of the study.

## ACKNOWLEDGEMENTS

We sincerely appreciate the participation of all patients involved in this study.

## FUNDING INFORMATION

Medical Research Center, University of Oulu, The Finnish Nursing Education Foundation.

## CONFLICT OF INTEREST STATEMENT

None declared.

## ETHICS STATEMENT

Approval for the study was obtained from each research centre and the Ethical Review Board of the University Hospital of Kuopio (Ref. 226/2015).

## Supporting information


Data S1:

## Data Availability

The data underlying this article will be shared upon a reasonable request to the corresponding author.
